# Spatiotemporal Variation Characteristics of Ecosystem Service Losses in the Agro-Pastoral Ecotone of Northern China

**DOI:** 10.3390/ijerph16071199

**Published:** 2019-04-03

**Authors:** Yuejuan Yang, Kun Wang, Di Liu, Xinquan Zhao, Jiangwen Fan, Jinsheng Li, Xiajie Zhai, Cong Zhang, Ruyi Zhan

**Affiliations:** 1 Institute of Grassland Science, China Agricultural University, Beijing 100093, China; yangyuejuan198907@163.com (Y.Y.); l628js@126.com (J.L.); zhaixiajie@cau.edu.cn (X.Z.); congzhang@cau.edu.cn (C.Z.); 13031130633@163.com (R.Z.); 2 Institute of Geospatial Information Science & Engineering, Hohai University, Nanjing 211100, China; liudi@x-gis.com.cn; 3 Northwest Plateau Institute of Biology, Chinese Academy of Sciences, Xining 810008, China; xqzhao@nwipb.cas.cn; 4 Key Laboratory of Land Surface and Simulation, Institute of Geographic Sciences and Natural Resources Research, Chinese Academy of Sciences, Beijing 100101, China; fanjw@igsnrr.ac.cn

**Keywords:** land-use conversions, agro-pastoral ecotone, human interventions, sustainable development

## Abstract

Being subject to climate change and human intervention, the land-use pattern in the agro-pastoral ecotone of Northern China has undergone complex changes over the past few decades, which may jeopardize the provision of ecosystem services. Thus, for sustainable land management, ecosystem services should be evaluated and monitored. In this study, based on Landsat TM/ETM data, we quantitatively evaluated the losses of ecosystem service values (ESV) in three sections of the agro-pastoral ecotone from 1980–2015. The results were as follows: (1) the main characteristic of the land conversions was that a large area of grassland was converted into cultivated land in the agro-pastoral ecotone; (2) on the spatial scale, the ESV losses of the agro-pastoral ecotone can be called an “inclined surface” in the direction of the northeast to southwest, and the northeastern section of the agro-pastoral ecotone lost more ESV than the middle and northwest sections (*p* < 0.05), on the temporal scale, the order of losses was 1990–2000 > 1980–1990 > 2000–2015; (3) the agro-pastoral ecotone lost more ESV, which was mainly due to four kinds of land conversion, which were grassland that was transformed into cultivated land, grassland transformed into unused land, grassland transformed into built-up areas, and cultivated land transformed into built-up areas; (4) although these land conversions were curbed after the implementation of protection policies at the end of the 1990s, due to reduced precipitation and increasing temperatures, the agro-pastoral ecotone will face a more severe situation in the future; and, (5) during the period of 1990–2015, the overall dynamic processes of increasing population gradually expanded to the sparsely populated pastoral area. Therefore, we believe that human interventions are the main cause of ecological deterioration in the agro-pastoral ecotone. This study provides references for fully understanding the regional differences in the ecological and environmental effects of land use change and it helps to objectively evaluate ecological civilization construction in the agro-pastoral ecotone of Northern China.

## 1. Introduction

Land use has profound ecological and environmental effects [1]. Land use alters the natural surface landscape and it simultaneously causes changes in ecosystem function and structure [2], thus playing a decisive role in maintaining ecosystem services [3,4]. Changes in land use patterns inevitably lead to changes in ecosystem services [5]. The ecological footprint [6], eco-environmental quality index [7], ecosystem services value [5], etc can characterize the effects of land use change. Choosing the ecosystem service values (ESV) that can be expressed in the form of monetary value to quantify the effects of land use change will alert relevant managers and decision-makers [8]. Costanza et al. (1997) evaluated global ESV by using a meta-analysis, determining the estimation principle and the scientific significance of ecosystem services [9]. Since then, further studies have reported that change in land-use/land-cover (LULC) strongly influences ecosystem services, such as rapid urbanization on the ecosystem service value [10], soil carbon [3], the surface terrestrial biogeochemical cycle [11,12], nutrient cycling [13], and recreation and aesthetic value [14]. Xie et al. (2008, 2015) formulated a table of ESV per unit area of different terrestrial ecosystems in China, which was concise and reliable and it has been widely used [8,15,16].

The major ecosystem services vary with different land use types. The LULC influences the main ecological process of ecosystem, including energy exchange, hydrological cycle, soil erosion, biogeochemical cycle, and so on, which further change the provision of ecosystem services. For instance, for cultivated land, its supply capacity for agricultural products is higher than its service competence of regulation, culture, and support. On the other hand, for natural grassland, its service capacity for regulation and support is higher than its product supply. Based on the evaluation of farmland ecosystem services, Ma et al. (2015) indicated that farmland ecosystems not only provided services for human beings, but they also consumed them (consumed economic investment, caused soil inorganic erosion, gave rise to pesticide pollution, etc.) [17]. Changes in the land-use pattern have an obvious influence on ecosystem services. Su et al. (2012) indicated that the landscape fragmentation, which was generated by the expansion of artificial land-use in the process of urbanization, might have a negative influence on ecosystem services [18]. Due to the preference of ecosystem services requirement, when involved in land-use administration, people often focus on one or a few types of ecosystem services, and thus unintentionally or intentionally affect the provision of other types of ecosystem service. For example, due to the blind pursuit of food, people occupy natural grassland to substantially expand the cultivated land, in this way, with the increment of grain output, the services of gas regulation, climate regulation, hydrology regulation, and soil conservation are dramatically weakened, i.e., the ESV losses is the value corresponding to the weakened services.

The agro-pastoral ecotone of Northern China (hereafter called the agro-pastoral ecotone) is a transition zone between agricultural cultivation and animal husbandry [19]. The agro-pastoral ecotone is the largest in area and the longest in spatial scale of all the typical ecotones in China [20,21]. Due to high migration inwards from other regions, grassland reclamation has been frequently undertaken since the end of the Qing dynasty in the nineteenth century [22], which neglects the natural laws of landscape ecological evolution. Although the agro-pastoral ecotone has some attributes of a transition zone in ecological and geographical aspects, it does not have typical transition zone characteristics: in the context of the unstable soil matrix, the land use structure is extremely irrational; its species diversity is not rich but is rather is extremely poor [23]; drought, rainstorms, hail, sandstorms, freezing, and other meteorological disasters are frequent [24]; and, the regional economy has a weak foundation, with 56.3% of the region belonging to national poverty counties where the available land resources and ecological carrying capacity are in sharp decline [22].

The agro-pastoral ecotone is pivotal for ecological security, social stability, and economic development; it is the origin of major Chinese rivers, a key area in resource exploitation, a multiethnic area, and an economic link between an agricultural zone and a pastoral area. Since the implementation of Chinese reform and opening up policies, under the dual influence of human activities and climate change, the agro-pastoral ecotone has experienced drastic changes in land use patterns [25,26]. Many scholars have studied the change in ESV that is caused by land use change in the agro-pastoral ecotone, for example, the Chifeng in Inner Mongolia [27], Ordos City [28], west Jinlin [8], the whole agro-pastoral ecotone [29], and at the national scale [15]. All of these studies showed that human interference had greatly changed the LULC, simultaneously affecting the ecosystem service provision. However, land use change has strong regional characteristics with different driving factors. The agro-pastoral ecotone has a large span from northeast to southwest, and there may be significant differences in regional precipitation, temperature, and population. However, there is a lack of relevant studies (simultaneously holistic and segmented) to assess the spatial and quantitative variation in the ESV losses that are caused by land use change in the agro-pastoral ecotone.

Therefore, the main objectives of this study are to: (1) analyze the spatiotemporal processes of land-use conversion in the three sections of the agro-pastoral ecotone; (2) quantitatively assess the ESV losses under the dual influences of population growth and climate change; and, (3) evaluate the ecological consequences of LULC change and their practical implications.

## 2. Materials and Methods

### 2.1. Study Area

**Figure 1:**
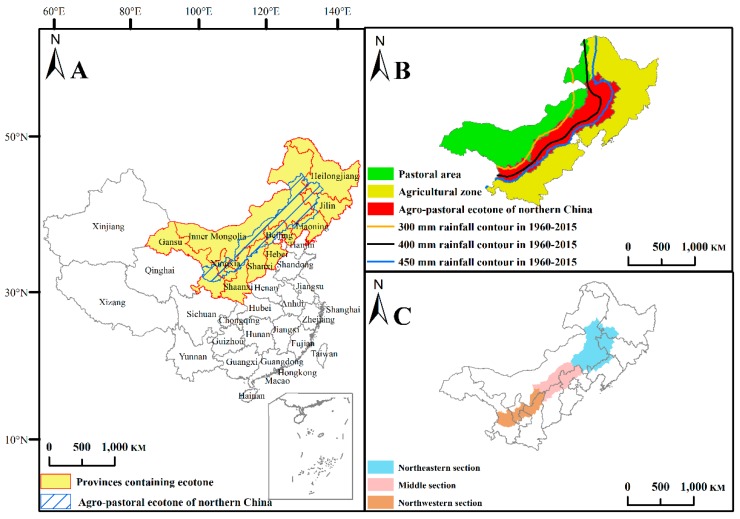
Geographical location of the agro-pastoral ecotone of Northern China (**A**), agricultural zone and pastoral area (**B**), and the three sections of the agro-pastoral ecotone (**C**).

**Figure 2:**
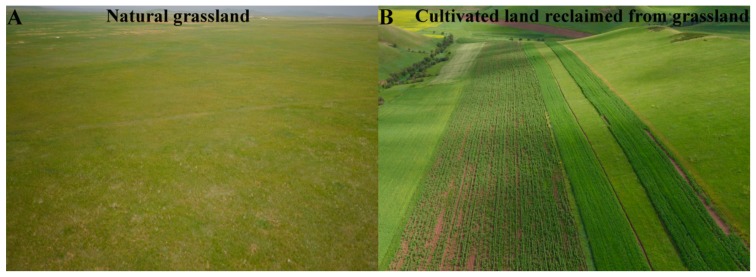
The main land use landscapes (**A**,**B**) in the agro-pastoral ecotone. Photos were taken in 2018. Photo A was taken in Zheng Lan Flag, Inner Mongolia. Photo B was taken in Horqin Right Front Banner, Inner Mongolia.

Zhao (1953) first proposed the concept of the agro-pastoral ecotone [19]. In recent years, the location of the agro-pastoral ecotone has been mainly redefined based on agro-meteorological factors [30,31,32]. In this study, the agro-pastoral ecotone is assumed to include nine provinces (Figure 1A), and it is mainly divided by the 400-mm rainfall contour, the agricultural zone to the east and south, and the pastoral area to the west and north (Figure 1B). The agro-pastoral ecotone has a total area of 654,000 km^2^, which mainly covers arid and semiarid regions, and the annual average precipitation is approximately 300–450 mm. The annual average temperature is approximately 2–8 °C, with a growing season from April to September, and there are 30–100 windy (>5 m/s) days of sand movement. Regarding the landscape, cultivated land and grassland are mutually staggered (Figure 2A,B). In this study, in order to fully understand the regional differences in the ecological and environmental effects of land use change, the agro-pastoral ecotone is divided into three parts: the northeastern section, middle section, and northwestern section (Figure 1C).

### 2.2. Assignment of Ecosystem Service Values (ESV)

There are some issues that need to be addressed when applying the framework of Costanza et al. (1997) to China. For instance, the total ESV likely reflect the level of ecosystem services in western countries; thus, the ESV are vastly overestimated for developing countries, such as China. However, the ESV for cultivated lands are thought to be underestimated [15,33]. When considering the actual situation in China, Xie et al. (2015) further developed the framework of Costanza et al. (1997), emphasizing the vital function of wetlands [34,35]. Permanent ice and snow, beach and shore, bottomland, and swampland were extracted from the original primary land-use types as a new primary LULC type, namely wetland (Table S1). Therefore, there were seven final primary LULC types on the raster maps (Figure S1). 

In this study, we used the framework of Costanza et al. (1997), however we employed ESV that were developed for China by Xie et al. (2015) (Table S2). According to the theory of ecosystem service evaluation, there are 21 types of land-use conversions that result in ecosystems losing ESV (Table 1).

### 2.3. Calculating ESV Losses

Based on the assessment model of Costanza et al. (1997), the equations used to evaluate ESV are as follows:

(1) $$V_{k}=\sum_{i=1}^{n}ESV_{ki}$$

(2) $$V_{j}=\sum_{i=1}^{n}ESV_{ji}$$

where, *V_k_* is the ESV of the LULC type *k*, *ESV_ki_* is the *i* type of *ESV_s_* of the LULC type *k*, *V_j_* is the ESV of the LULC type *j*, and *ESV_ji_* is the *i* type of ESV of the LULC type *j*. If *V_k_* > *V_j_*, then:

(3) $$ESV_{loss}=\Delta A*(V_{k}-V_{j})$$

(4) $$ESV_{t}=\frac{\sum ESV_{loss}}{A}$$

where *ESV_loss_* is the loss of ESV when the LULC type *k* transformed into the LULC type *j*; Δ*A* is the corresponding land-use transformation area, *ESV_t_* is the ESV losses per unit area for all of the land-use transformations in the research region, and *A* is the research region area. To make the agricultural zone, agro-pastoral ecotone, and pastoral area comparable, the ESV losses that are mentioned below refer to *ESV_t_*.

### 2.4. Data Sources

We utilized LULC maps with 30 m grids from 1980, 1990, 2000, and 2015. The Data Center for Resources and Environmental Sciences, Chinese Academy of Sciences provided these maps. Based on Landsat TM/ETM data, the images were geometrically corrected and geo-referenced. Additionally, an outdoor survey and random sample check were performed, which confirmed that the average accuracy of the interpretation of LULC changes exceeded 90% [36,37]. The Institute of Geographic Sciences and Natural Resources Research, Chinese Academy of Sciences provided the annual precipitation, average temperature, and population data with 1 km grids.

Furthermore, we used a dynamic ESV assessment method to validate our results. The net primary productivity (NPP) was derived while using the improved Carnegie–Ames–Stanford approach (CASA) model [35,38]. The spatiotemporal regulation factor of soil conservation was derived from the Revised Universal Soil Loss Equation (RUSLE), which is the most widely used equation in the world for estimating soil erosion.

### 2.5. Data Statistical Analysis

The trend analysis of the ESV losses was implemented by moving weighted trend surface analysis [39,40], which was calculated in MATLAB 2014a (MathWorks, Natick, MA, USA). Appendix A provides the details of the process. The Mann-Kendall test was used to analyze the shifting trends of annual precipitation, average temperature, and population. Appendix B provides the details of the method. The directional distributions of population patterns, as performed by standard deviation ellipse analysis [41,42], were analyzed in ArcGIS 10.2 (Esri, Redlands, CA, USA). More detailed information on the calculation process can be found in Appendix C.

Partial least squares-discriminant analysis (PLS-DA), which is a multivariate statistical method, is a combination of partial least squares regression and the discrimination analysis for classification tasks [43,44]. PLS-DA can reduce the influence of multicollinearity among indices, which makes the analysis of the relationship between the independent and dependent variables more reasonable [45]. Moreover, the variable importance in projection (VIP) score reflects the influence of the independent variable on the dependent variable [46,47], and its equation is: $VIP_{j}=\sqrt{\frac{p\sum_{h=1}^{m}Rd(Y;t_{h}){\omega^{2}}_{hj}}{Rd(Y;t_{1},\cdots ,t_{m})}}$, where *VIPj* represents the *VIP* value of the *j*-th index, *p* is the number of selected index variables, *m* is the extracted PLS principal component number, *Rd*(*Y*; *t_h_*) represents the explanatory power of axis *t_h_* on *Y*, ${\omega^{2}}_{hj}$ is the *j*-th weight of axis $\omega_{h}$, and *Rd*(*Y*; *t*_1_, …, *t_m_*) represents the cumulative explanatory power of axis *t*_1_, …, *t_m_* on *Y*. Herein, PLS-DA was used to differentiate the ESV losses at the spatiotemporal scale, which was performed in the SIMCA 14.1 software (Umetrics, Umeå, Västerbotten, Sweden). 

## 3. Results

### 3.1. Spatiotemporal Processes of Land-Use Conversions in the Agro-Pastoral Ecotone

**Figure 3:**
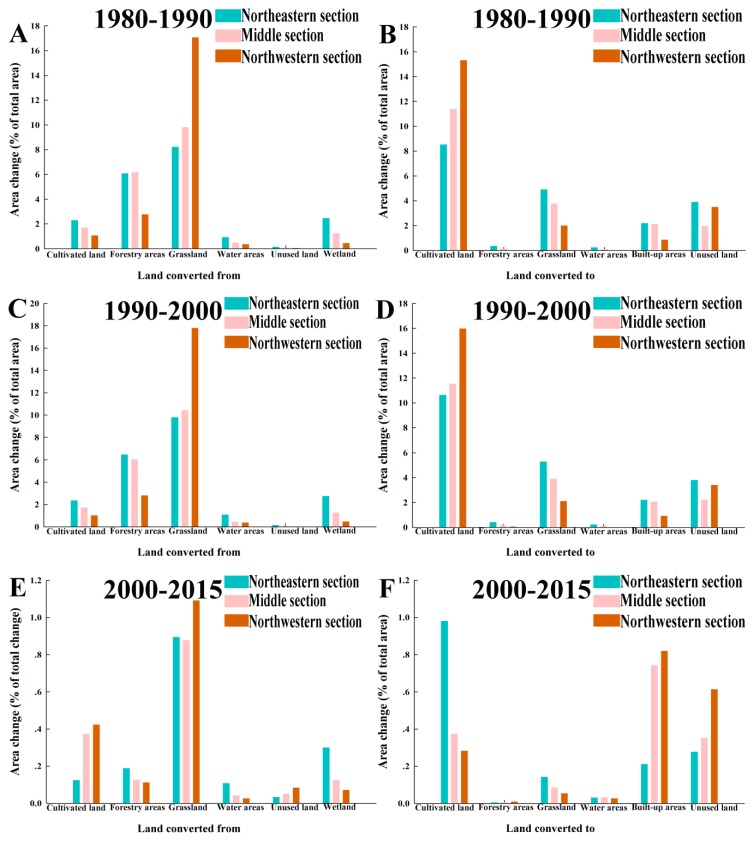
Land-use conversions in the agro-pastoral ecotone in 1980–1990 (**A**,**B**), 1990–2000 (**C**,**D**), and 2000–2015 (**E**,**F**).

For “land converted from” (Figure 3A,C,E), during 1980–2015, the area change of grassland in the northwestern section was the greatest among all of the land-use conversions, with values of 17.05, 17.78, and 1.09% for 1980–1990, 1990–2000, and 2000–2015, respectively. The grassland of the middle section in 1980–1990 and 1990–2000 followed, with area changes of 9.80 and 10.41%, respectively. In addition to the grassland of the three sections, the cultivated land underwent a significant transformation in 2000–2015. 

For “land converted to” (Figure 3B,D,F), the overall characteristic was that most land types were transformed into cultivated land. During 1980–1990 and 1990–2000, the area change of cultivated land in the northwestern section was the greatest, presenting values of 15.30 and 15.96%, respectively. The next greatest change was in the cultivated land of the middle section in 1980–1990 and 1990–2000, with values of 11.37 and 11.53%, respectively. In addition to the cultivated land, the built-up areas and the unused land of the three sections showed a significant increasing trend in 2000–2015. The above results indicate opposing trends in grassland and cultivated land in the agro-pastoral ecotone, which may completely reflect the evolution process of the agro-pastoral pattern.

**Figure 4:**
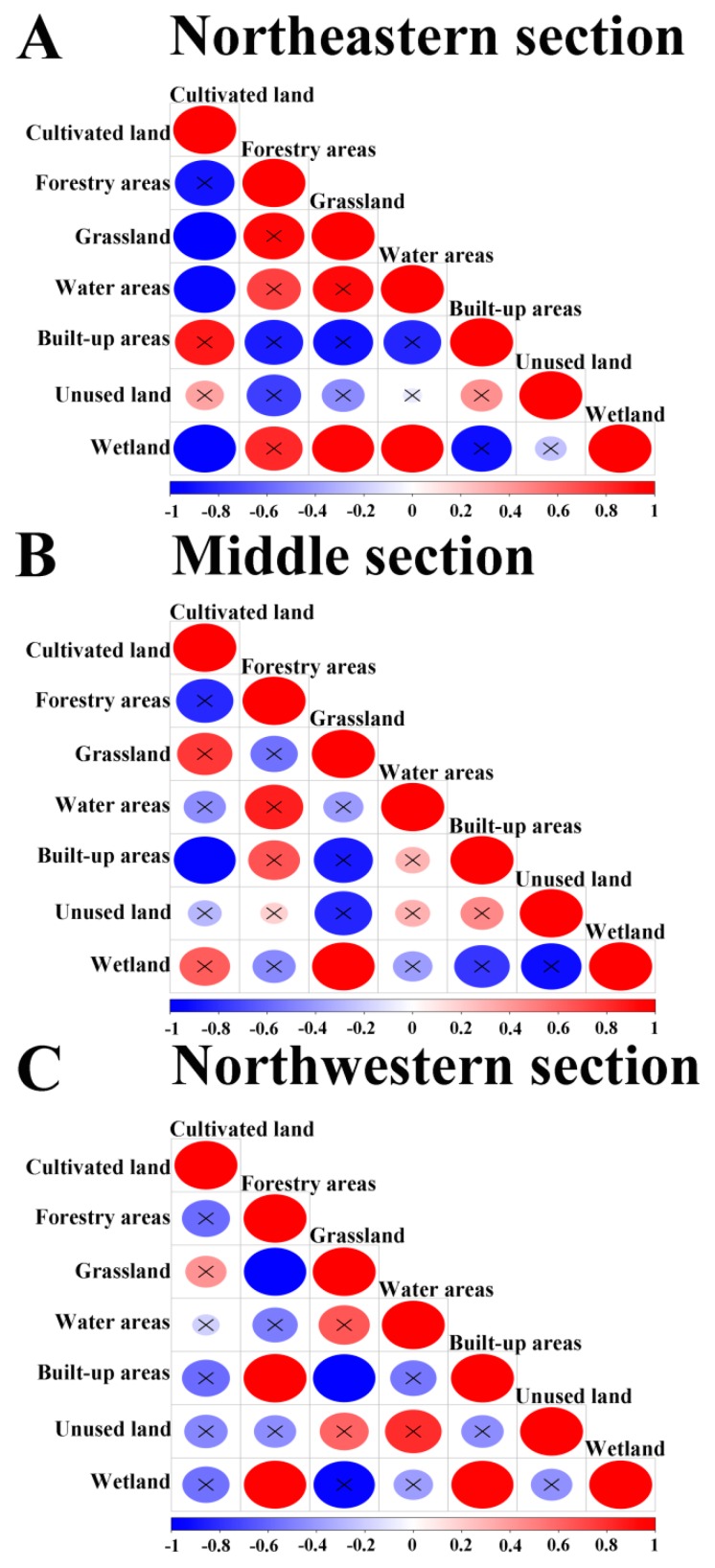
Correlation matrix of land-use proportions in the northeastern section (**A**), middle section (**B**), and northwestern section (**C**) of the agro-pastoral ecotone from 1980 to 2015. Note: × indicates nonsignificance (*p* > 0.05). X-axes denote the correlation coefficient.

Correlation is the relationship between the values of two variables (or indicators) that are professionally related to each other to some extent, and the values of the variables either increase synchronously or one increases and the other decreases. The correlation matrix, which is also called the correlation coefficient matrix, is composed of the correlation coefficients among the columns of the matrix. That is, the element of the *i*-th row and *j*-th column in the correlation matrix is the correlation coefficient of the *i*-th and *j*-th column of the original matrix. In the northeastern section, the proportion of cultivated land was negatively correlated with grassland, water areas, and wetland, with statistically significant (*p* < 0.05) correlation coefficients that are lower than −0.8 (Figure 4A), while the proportion of wetland was statistically significantly positively correlated with grassland and water areas. In the middle section of the agro-pastoral ecotone, the proportion of cultivated land was strongly negatively correlated with the built-up areas, and there was a statistically significant positive correlation between the proportion of grassland and wetland (Figure 4B). In the northwestern section, the proportion of grassland was significantly negatively correlated (*p* < 0.05) with forestry areas and built-up areas, with correlation coefficients lower than −0.8. The built-up areas were significantly positively correlated with forestry areas and wetlands, with correlation coefficients that are greater than 0.8. The forestry areas and wetlands were significantly positively correlated (Figure 4C).

### 3.2. Analysis of ESV Losses in the Agro-Pastoral Ecotone

**Figure 5:**
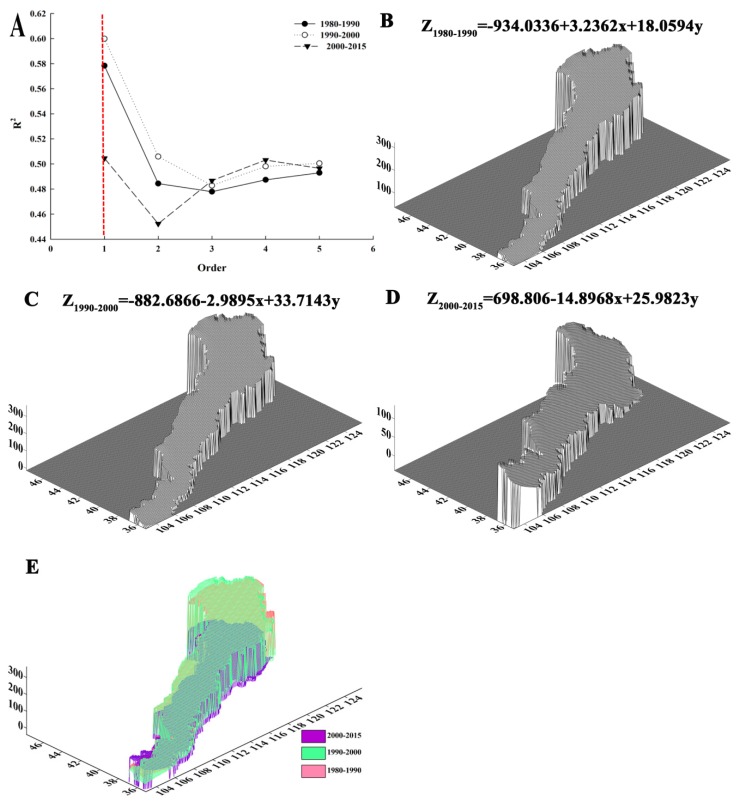
Optimal model selection (**A**), quadratic trend surface model in 1980–1990 (**B**), 1990–2000 (**C**), 2000–2015, (**D**) and comparison of the three periods (**E**). Note. The projection of the trend surface is from the orthogonal view, rotated 45° and inclined 30°; the bigger the value is, the more ecosystem service values (ESV) the region lost. Note: The red dotted line (Figure 5A) indicates the goodness of fit of ESV losses in three time periods when the order is equal to 1. Z, the ESV losses ($/hm^2^). R^2^, the the goodness of fit.

The trend surface polynomial analysis method simulates the distribution of the geographical elements in space, displaying the variation trend of elements in geographical space. Generally, the higher the polynomial order, better the fitting effect of trend surface near data collection point will be. However, when the order is excessively high, the interpolation and extrapolation properties will significantly worsen, and therefore the order should not exceed five in the trend surface analysis [48]. Therefore, to select the optimal model, we simulated the ESV losses with five orders (Figure 5A). The goodness of fit (R^2^) of the first order trend surface model, which was the highest of all orders, was 0.58, 0.60, and 0.51 for the periods 1980–1990, 1990–2000, and 2000–2015, respectively. Thus, it was used to assess the trend of ESV losses in response to land-use conversions. The first order trend surface, which delineated the overall distribution of ESV losses, was similar to an “inclined surface”. Figure S2 of Supplementary Files provides the schematic diagram of the first order trend surface. The losses in the northeastern section are the highest, followed by the middle section and the northwestern section (Figure 5B–D). On the temporal scale, the order of ESV losses was 1990–2000 > 1980–1990 > 2000–2015 (Figure 5E). The parameters of the first order trend surface model were statistically significant (*p* < 0.05), and the estimated regression equations fitted well with the measured values (R^2^ > 0.5), which were high for the cross-section data (Table S3). 

Additionally, we used another method to verify the reliability of our results, the spatiotemporal dynamic equivalence factor method, to assess the ESV losses in 2010–2015 (Appendix D and Figure S3). In the spatiotemporal dynamic equivalence factor method, the equivalence factor table of ESV is revised and supplemented to establish the spatiotemporal dynamic evaluation methods for different ecosystem types. The result also showed that it was a first order trend surface model. Furthermore, when compared with the method of equivalence factor per unit area that was used in this study, the spatiotemporal dynamic equivalence factor method indicated that the three sections lost more ESV (Figure S3).

**Figure 6:**
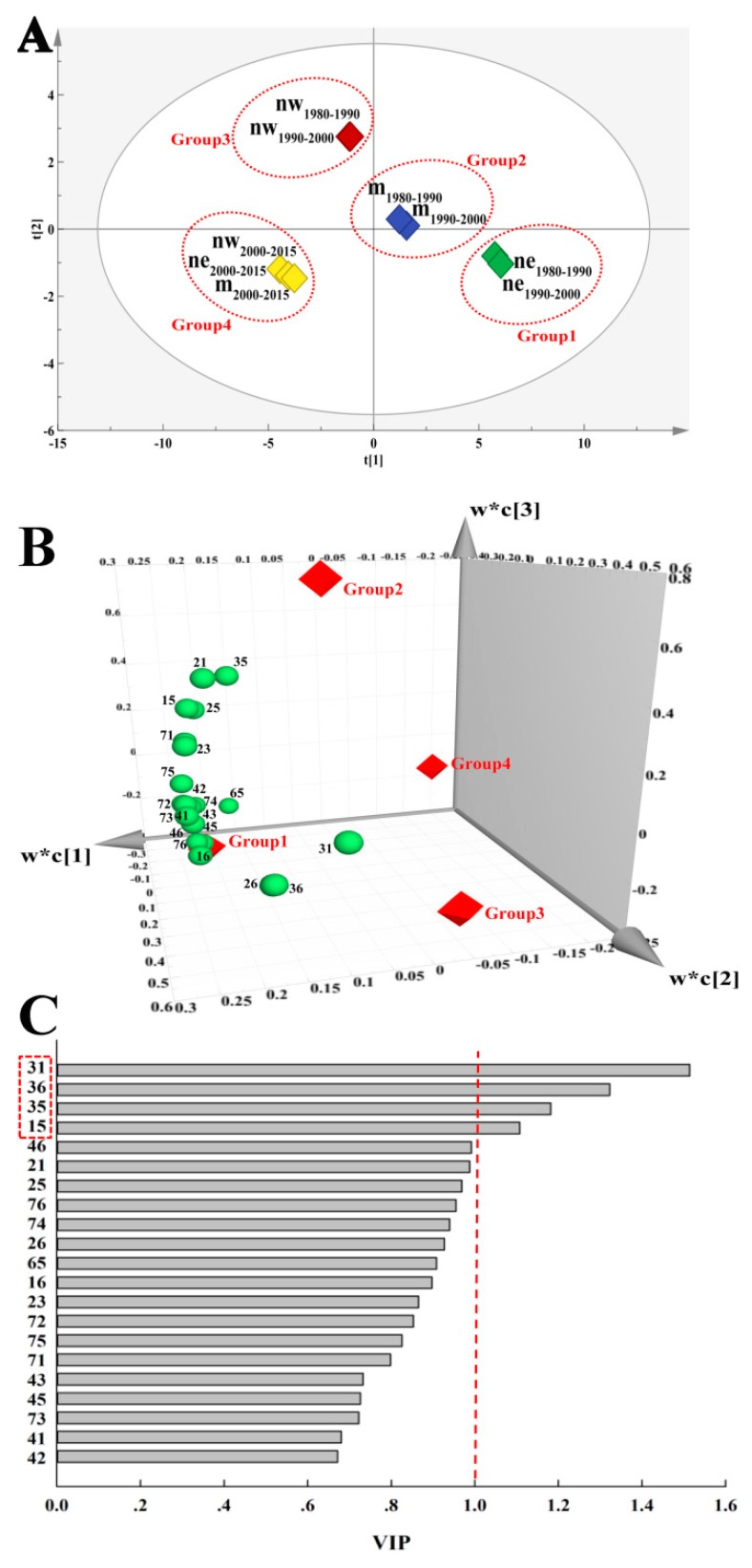
Partial least-squares discriminant analysis (PLS-DA) score scatter plot (**A**), loading scatter three-dimensional (3D) plot (**B**), and variable importance in projection (VIP) (**C**). Note: ne, northeastern section; m, middle section; nw, northwestern section. t[1], the score of the first principal component. t[2], the score of the second principal component. w*c[1], the first loading coefficient. w*c[2], the second loading coefficient. w*c[3], the third loading coefficient. 15, cultivated land transformed into built-up areas; 16, cultivated land transformed into unused land; 21, forestry areas transformed into cultivated land; 23, forestry areas transformed into grassland; 25, forestry areas transformed into built-up areas; 26, forestry areas transformed into unused land; 31, grassland transformed into cultivated land; 35, grassland transformed into built-up areas; 36, grassland transformed into unused land; 41, water areas transformed into cultivated land; 42, water areas transformed into forestry areas; 43, water areas transformed into grassland; 45, water areas transformed into built-up areas; 46, water areas transformed into unused land; 65, unused land transformed into built-up areas; 71, wetland transformed into cultivated land; 72, wetland transformed into forestry areas; 73, wetland transformed into grassland; 74, wetland transformed into water areas; 75, wetland transformed into built-up areas; 76, wetland transformed into unused land.

According to the score scatter plot (Figure 6A), the goodness of fit (R^2^ = 0.92) demonstrated a high discriminative ability of PLS-DA. The three regions were clearly divided into four groups in the three periods: Group 1, northeastern section in 1980–1990 and 1990–2000; Group 2, middle section in 1980–1990 and 1990–2000; Group 3, northwestern section in 1980–1990 and 1990–2000; and, Group 4, northeastern section, middle section and northwestern section in 2000–2015. As shown in Figure 6B, Groups 1 and 2 lost more ESV, as they were close to more land-use conversions, while Groups 3 and 4 were far away from these land-use conversions and they thus did not lose too many ESV. 

Additionally, an important discriminative variable usually has a VIP score that is greater than 1. As shown in Figure 6C, the VIPs of grassland transformed into cultivated land, grassland transformed into unused land, grassland transformed into built-up areas, and cultivated land transformed into built-up areas were greater than 1, which meant that the classifications of the three regions were distinct from each other on the spatiotemporal scale.

To show the areas of ESV change, the ESV losses have been visualized onto maps (Figure S4). The results were consistent with the PLS-DA on a time scale. During 1980–1990 and 1990–2000, the whole ecotone lost more ESV, however the ESV losses also significantly decreased in 2000–2015. The regions that lost most ESV in 2000–2015 were as follows: In the northeastern section: Tailai County, Zhenlai County, Daan City, Kailu County, Horqin Left Middle Banner, etc.; in the middle section: Horinger County, Togtoh County, Tumd Left Banner, Jungar Banner, etc.; and, in the northwestern section: Ejin Horo Banner, Shenmu County, Wushen County, etc.

### 3.3. Variance Analysis and Trend Analysis of Precipitation, Temperature and Population in the Agro-Pastoral Ecotone

It is necessary to analyze their regional differences and changing trends due to the significant influence of climate and human factors on LULC and ESV. Several analyses are made as follows: variance analysis (precipitation, temperature, and population) in the three sections of agro-pastoral ecotone, trend analysis of annual averages (precipitation, temperature, and population) in the three sections of agro-pastoral ecotone, interdecadal shift analysis of 400 mm rainfall contour, and standard deviation ellipse of population.

**Figure 7:**
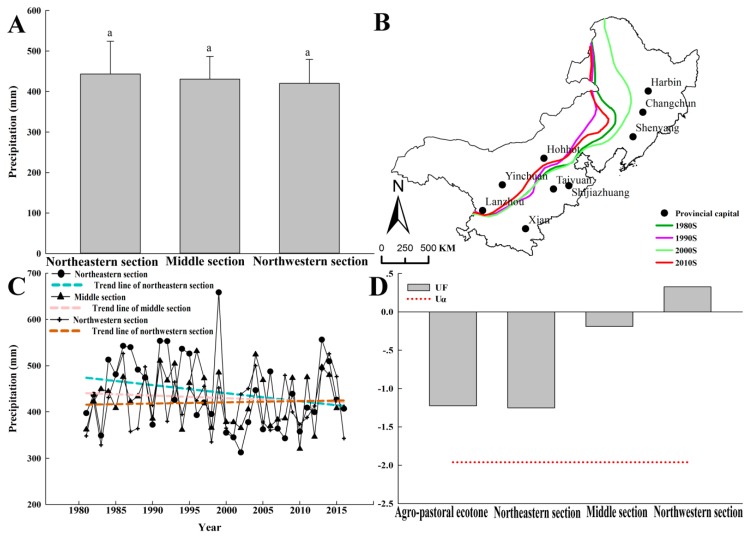
Variance analysis of precipitation (**A**), interdecadal shift of the 400 mm rainfall contour (**B**), average annual precipitation from 1980 to 2015 (**C**), and Mann-Kendall trend analysis of average annual precipitation (**D**). Note: *UF*, the value used to evaluate the statistically significant trend; Significance level, α = 0.05; *U_α_* = ±1.96. The same are used below.

Although there were no significant differences in the average annual precipitation among the three regions (Figure 7A), the 400 mm rainfall contour strongly fluctuated during 1980–2015, especially in the northeastern and middle section of the agro-pastoral ecotone (Figure 7B). The average annual precipitation had decreasing trend lines in the three regions (Figure 7C), i.e., the agro-pastoral ecotone tended towards aridness. However, the Mann-Kendall test showed that the trend of precipitation reduction was not significant (|*UF*| < 1.96) (Figure 7D).

**Figure 8:**
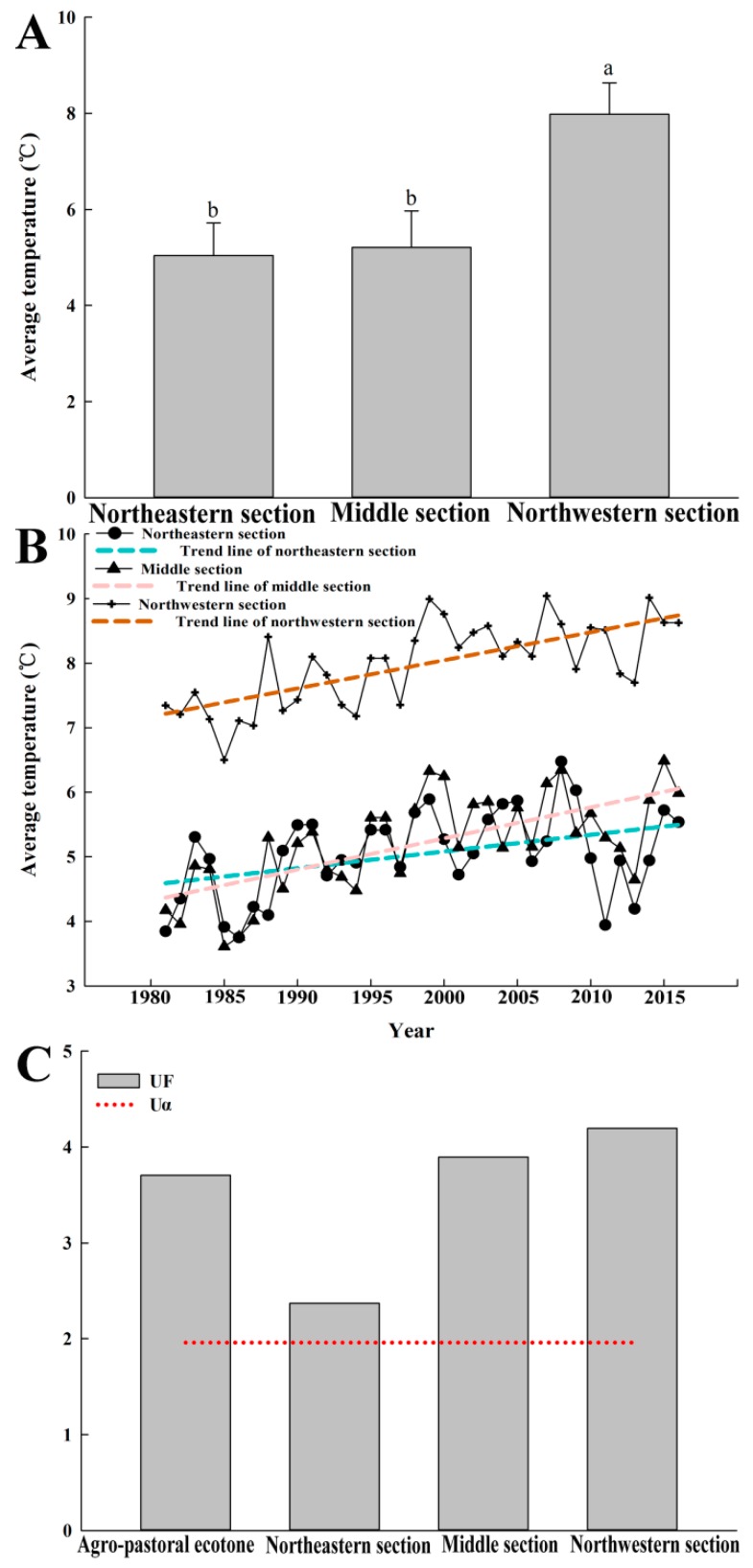
Variance analysis of air temperature (**A**), average annual temperature from 1980 to 2015 (**B**), and Mann-Kendall trend analysis of average annual temperature (**C**) for the agro-pastoral ecotone.

There was no difference in the average temperatures between the northeastern section and the middle section of the agro-pastoral ecotone, however the temperatures of these two sections were significantly different from those of the northwestern section (Figure 8A). The average temperature had increasing trend lines in all three regions (Figure 8B), i.e., the agro-pastoral ecotone tended to have a higher temperature. The Mann-Kendall test showed that this increasing trend was significant (|*UF*| > 1.96) (Figure 8C). Furthermore, when compared with the overall agro-pastoral ecotone, this trend was more obvious in the middle section and the northwestern section (Figure 8C).

**Figure 9:**
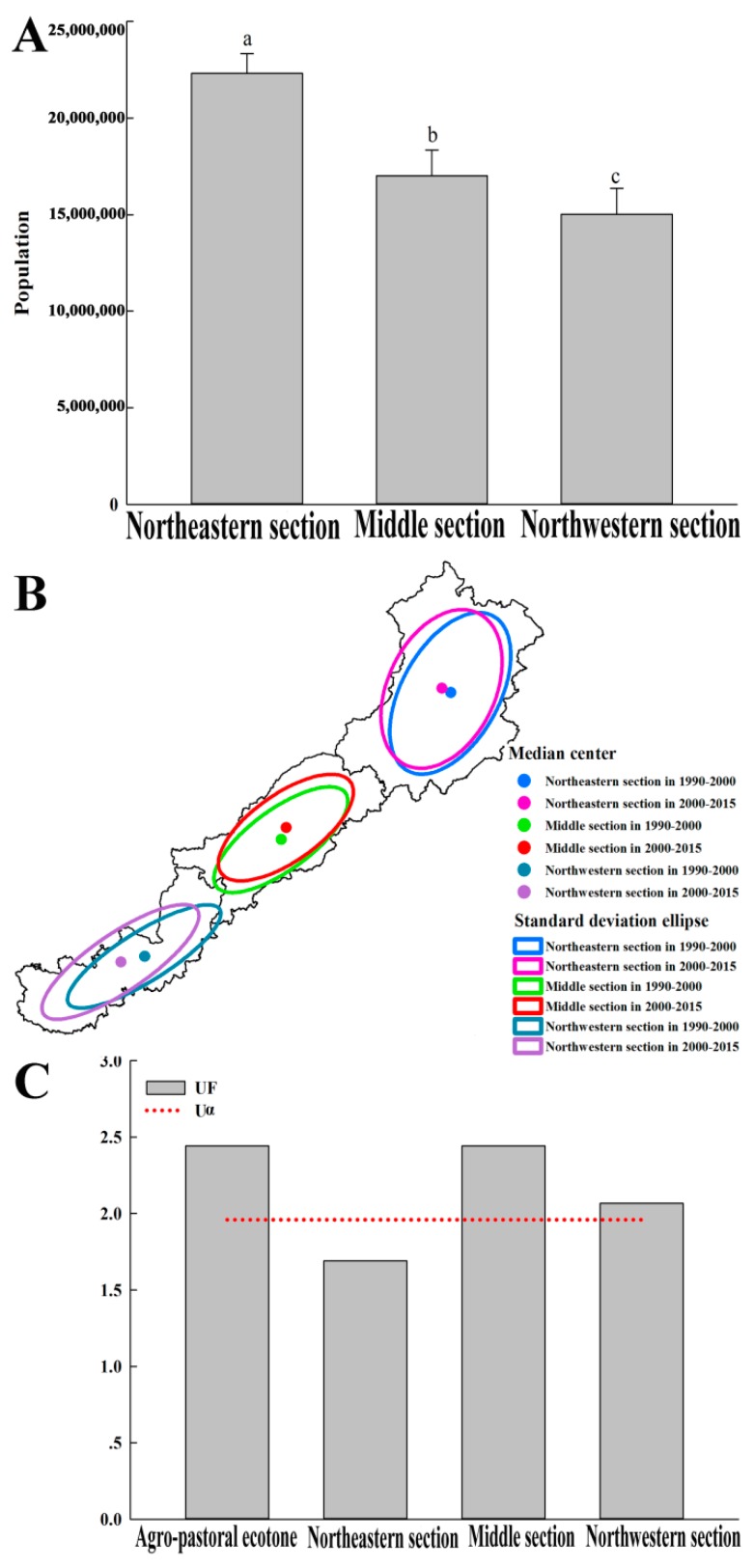
Variance analysis of population (**A**), standard deviation ellipse of increasing population (**B**), and Mann-Kendall trend analysis of population (**C**).

As shown in Figure 9A, there were significant differences in the population among the three sections. Standard deviation ellipse analysis further analyzed the changes in population. During the period of 1990–2015, the overall dynamic processes of increasing population gradually expanded to the sparsely populated pastoral area (Figure 9B). The Mann-Kendall test showed that the increasing trend of population was significant in the middle section and the northwestern section (|*UF*| > 1.96) (Figure 9C). Furthermore, when compared with the trend in the overall agro-pastoral ecotone, the trend was more obvious in the middle section.

## 4. Discussion

### 4.1. Human Interferences Led to ESV Losses in the Agro-Pastoral Ecotone Losing

In semi-arid areas, grassland husbandry is the main land use type. It is a common practice in the United States of America (USA), Australia, and other semi-arid areas. However, the agro-pastoral ecotone of Northern China, which also contains semi-arid areas, is dominated by rain-fed agriculture. This agro-pastoral ecotone is a special ecological–socia–leconomic system that was formed by the large-scale arrival of immigrants and extensive reclamation. The three sections of the agro-pastoral ecotone have undergone drastic LULC change, with a sharp increase in cultivated land that is mainly caused by the overexploitation of traditional natural grassland, since the 1980s. The consequences of this were that the ecotone lost more ESV. Terrestrial ecosystems not only continuously provide food, fiber, fuel, and other products for humans, but also simultaneously supply public goods that cannot be delivered by the market, such as climate regulation, hydrology regulation, waste regulation, soil conservation, and aesthetic landscape provision, bringing substantial welfare to human [8,9,49]. This could cause ecosystems to have high environmental load and low sustainability, purely for the purpose of economic development and personal needs [23,50]. In this study, the results of the moving weighted trend surface analysis showed that the ESV losses pattern of the agro-pastoral ecotone was similar to an “inclined surface”, i.e., the northeastern section of the ecotone lost more ESV than the other sections. This virtual “surface” is an expression of ecological vulnerability on a macro scale that is more sensitive to external disturbances [51,52]. The surface, which is a visible manifestation of strained human-land relations, was mainly formed by the long-term improper use of land resources and it provides a quantitative description of ESV losses.

The results show that the total population of the northeastern section of the agro-pastoral ecotone was significantly larger than that of the middle section and the northwestern section. Additionally, based on the results of the standard deviation ellipse, it was found that the overall population dynamic process gradually expanded to the sparsely populated pastoral area. Thus, rapid population growth may have been the main driver behind these land conversions. This finding is consistent with the analysis for Northern China and the Loess Plateau by Liu et al. (2014). It is noteworthy that the Mann-Kendall trend analysis indicated that the population showed a significantly increasing trend in the middle section and the northwestern section (|*UF*| > 1.96), while the population growth in the northeastern section was not significant (|*UF*| < 1.96).

By quantitative analysis of PLS-DA in this study, we have shown that land transformations (grassland transformed into cultivated land, grassland transformed into unused land, grassland transformed into built-up areas, and cultivated land transformed into built-up areas) were decisive land conversions that led to a vast reduction in ESV. Due to the restraint of water, soil fertility, and other factors, crop productivity in those areas was low, which led to a vicious circle of population growth—land reclamation—ecosystem deterioration—further reclamation. The grain yields were able to meet people’s needs in years of plentiful rainfall; however, they dramatically decreased or fell to zero in drought years [36,53]. To acquire enough food, people cannot help but constantly reclaim new grassland, simultaneously abandoning infertile cultivated land [24,54], which gradually transforms into unused land (e.g., sandy land, salina, and bare soil). By analyzing the emergy differences among the terrestrial ecosystems, from the perspective of the environment and productivity, it has been proven that rain-fed artificial grassland has a higher development potential in the agro-pastoral ecotone [23]. Moreover, under urbanization and increasing population, urban buildings and rural settlements have encroached onto the grasslands of the agro-pastoral ecotone [55]. The ecotone is also a key area of resource exploitation, as it is rich in mineral resources [22]. Considerable amounts of grassland have been transformed into built-up areas, including large industries, oil fields, salt fields, quarries, roads, and other specialized land uses. However, most of the regions in the agro-pastoral ecotone were national poverty counties, where economic development came at the cost of the ecological environment [56]. Extensive management and long-term unrestricted mining have caused the agro-pastoral ecotone to lose substantial ESV [15,26].

### 4.2. Natural Factors that Have Led to ESV Losses in the Agro-Pastoral Ecotone

Furthermore, as an important ecological boundary, any small movement of the 400 mm rainfall contour will bring about great changes in an ecosystem [57]. In this study, we find that precipitation will be probably diminished in the future in the agro-pastoral ecotone. Additionally, the temperatures of the three regions of the ecotone have obviously increasing trends, which indicates that the ecotone will have to face a warm and dry climate, a more severe situation, in the future. Hydraulic erosion and wind erosion coexist in the agro-pastoral ecotone. These two types of soil erosion promote mutually, interlacing on the temporal scale and superimposing on the spatial scale. Therefore, the erosional energy of the agro-pastoral ecotone is significantly higher than that of the region of mainly hydraulic erosion (agricultural zone) and that of the region of mainly wind erosion (pastoral area) [22]. In the spring, when the land surface is thawed with sparse vegetation, sand movement is extremely serious, as there is faster soil water evaporation in dry years. It has been proven that the agro-pastoral ecotone is a source of sandstorms in many areas, especially in Beijing–Tianjin [25,58]. Based on the spatial distribution of the soil texture, the soil in the agro-pastoral ecotone is mainly sandy soil [20,59,60]. A low coverage, weak soil capacity and low water conservation capacity characterize the fragile surface structure, so that even minor disturbances can cause damage to the ecosystem. Moreover, the annual precipitation in the agro-pastoral ecotone has high variability. The annual precipitation can be as high as 500–600 mm, however it can also be less than 200 mm [61]. Precipitation is generally concentrated in the summer, with 60–70% of the yearly amount occurring in June–August, when the area is prone to rainstorms and it has extremely low water-use efficiency. Groundwater is continuously pumped for irrigation, creating funnel regions and shrinking wetland [62]. Natural conditions are inappropriate for farming in the agro-pastoral ecotone.

Based on the above, we conclude that the agro-pastoral ecotone is affected by a combination of natural factors and human interference, with the former determining its potential ecological fragility and the latter resulting in a truly fragile ecosystem.

### 4.3. Ecological Protection Policies Implemented in the Agro-Pastoral Ecotone

Long-term eco-restoration projects are critical in sustainable ecological and economic development [31,63,64]. Since the end of the 1990s, China has initiated specific ecological protection polices, such as the Grain for Green Project, the Beijing–Tianjin Dust Storms Sources Control Project, Grassland Ecological Protection Subsidy Incentives, and the Grain for Forage Project, with remarkable effects. Through the moving weighted trend surface analysis and PLS-DA that were conducted in this study, we found that the order for the ESV losses was 1990–2000 > 1980–1990 > 2000–2015 in the agro-pastoral ecotone. Based on these policies, it may be concluded that proper strategies can restore ecosystem functions and improve ecosystem services in limited resources.

### 4.4. Validity and Limitations of This Study

Due to the difficulty in estimating the value from the non-marketed components of ecosystem services, the existing socioeconomic statistical system does not fully account for ESV. Although many previous studies [9,35,65,66,67,68] have evaluated the valuation of ecosystem services, it is still difficult to identify, quantify, and monetarize ESV. There is still no unified and complete set of scientific assessment methods for ESV. For most of the studies, the obvious barrier is a lack of relevant data in the assessment of ESV, which leads to a rough evaluation result being obtained [69,70,71]. In some small areas, the data that were collected from field observation and survey were able to meet their requirements; however, for large areas, if we still used the same methods to acquire data, except for longer periods and higher costs, large-scale models might abridge some indicators due to the shortage of data, which often caused the final results to only show the average level of the whole changing trend [70]. Reviewing the research literatures on ecosystem services, Martínez-Harms and Balvanera (2012) indicated that the studies of ESV mainly focused on the regional- and country-scale, and the service of regulation was the most studied type [71]. When compared to original data derived from field surveys and experimental observations, secondary data, such as remote sensing data and social economic data, were the more frequently used.

The method of equivalence factor per unit area used in this study is mainly applied to decision-making when estimating ESV at broad regional or national scales [15], as it allows for a quick assessment. More micro-level mechanisms were not studied due to a lack of reliable methods and relevant data. To verify reliability, we used another method, the spatiotemporal dynamic equivalence factor method, to assess the ESV losses in 2010–2015. The dynamic assessment method was consistent with our results. Since our method reflects the large-scale average level compared with the spatiotemporal dynamic equivalence factor method, the inclined first order trend surface from northeast to southwest is underestimated. Given the substantial uncertainties that are involved, we may never have an accurate evaluation of ESV losses [35]. However, this study may be a starting point in emphasizing the potential threat of continuing to squander ecosystem services.

## 5. Conclusions

Satellite-based data can substantially assist in the monitoring of large-area landscape changes and in analyzing the effects of landscape changes on ESV losses. In this study, the spatiotemporal process of land-use conversions and their impacts on ecosystem services were investigated in three sections of the agro-pastoral ecotone of Northern China. Significant expansion of cultivated land and reduction in grassland area characterized the trend of land conversion during 1980–2000, whereas the trend was effectively controlled during 2000–2015. When compared with the middle section and the northwestern section of the agro-pastoral ecotone, the irrational land use patterns caused more ESV losses in the northeastern section. In terms of ecosystem service provisions, the agro-pastoral ecotone can be called an “inclined surface” in the direction of northeast to southwest. We believe that human beings play a decisive role in the unique agro-pastoral ecotone of Northern China. Based on the assessment of ecosystem services in the northeastern section, middle section, and northwestern section, long-term restoration projects are crucial in sustainable development in the agro-pastoral ecotone. However, with the reduction in precipitation and increasing temperatures, it should be noted that the risk of ESV losses is still severe. The process of land conversion in the agro-pastoral ecotone requires sustained attention. This study provides some preliminary findings, which could be helpful in improving the understanding of ESV change in response to regional land-use conversions in the agro-pastoral ecotone. Due to the complexity of sustainable land management, it may be a starting point, and more needs to be done in the future.

## Figures and Tables

**Table 1:** Land-use transfer matrix leading to losses of ecosystem service values (ESV).

	1 Cultivated Land	2 Forestry Areas	3 Grassland	4 Water Areas	5 Built-Up Areas	6 Unused Land	7 Wetland
1 Cultivated land					15	16	
2 Forestry areas	21		23		25	26	
3 Grassland	31				35	36	
4 Water areas	41	42	43		45	46	
5 Built-up areas							
6 Unused land					65		
7 Wetland	71	72	73	74	75	76	

Note: 15, cultivated land transformed into built-up areas; 16, cultivated land transformed into unused land; 21, forestry areas transformed into cultivated land; 23, forestry areas transformed into grassland; 25, forestry areas transformed into built-up areas; 26, forestry areas transformed into unused land; 31, grassland transformed into cultivated land; 35, grassland transformed into built-up areas; 36, grassland transformed into unused land; 41, water areas transformed into cultivated land; 42, water areas transformed into forestry areas; 43, water areas transformed into grassland; 45, water areas transformed into built-up areas; 46, water areas transformed into unused land; 65, unused land transformed into built-up areas; 71, wetland transformed into cultivated land; 72, wetland transformed into forestry areas; 73, wetland transformed into grassland; 74, wetland transformed into water areas; 75, wetland transformed into built-up areas; 76, wetland transformed into unused land.

## Supplementary Materials

The following are available online at https://www.mdpi.com/1660-4601/16/7/1199/s1. Figure S1: Land-use/land-cover (LULC) of the study area in 2015. Figure S2: Schematic diagram of the first order trend surface. Figure S3: The result of evaluating the ESV losses in 2010–2015. Figure S4: Spatial distribution of hotspots of ESV losses in 1980–1990 (**A**), 1990–2000 (**B**) and 2000–2015 (**C**). Table S1: LULC classification. Table S2: Equivalence factor per unit area of ESV in China ($/ha × year based on the 2007 value of USD). Table S3: Parameters of the first order trend surface fitting.

## Appendix A. Moving Weighted Trend Surface Analysis

The trend surface analysis, based on the least sum of squares, is a multivariate polynomial regression analysis that can be used to fit mathematical surfaces of increasing complexity to a variable distribution on a map. The expressions of the polynomial trend surfaces are

(A1) $$Z_{i}\left(x_{i},y_{i}\right)={\hat{Z}}_{i}(x_{i},y_{i})+e_{i}$$

(A2) $${\hat{Z}}_{i}\left(x_{i},y_{i}\right)=a_{0}+a_{1}x_{i}+a_{2}y_{i}+a_{3}x_{i}y_{i}+a_{4}x_{i}^{2}+a_{5}y_{i}^{2}+\cdots a_{p}y_{i}^{n}$$

where $Z_{i}\left(x_{i},y_{i}\right)$, ${\hat{Z}}_{i}\left(x_{i},y_{i}\right)$, and $e_{i}$ are the observed, trend, and residual values of variable Z at coordinate $\left(x_{i},y_{i}\right)$, respectively, and $a_{0}$ to $a_{p}$ are the polynomial coefficients.

The variables in Equation (A2) are written in matrix form:

(A3) $${\hat{Z}}_{i}\left(x_{i},y_{i}\right)=AX$$

Based on the least-squares theory, the residual sum of squares (*Q*) between the observed value $Z_{i}$ and the trend value ${\hat{Z}}_{i}$ should be minimized to achieve a “best fit”. Thus:

(A4) $$Q={\sum_{i=1}^{n}\left[Z_{i}(x_{i},y_{i})-{\hat{Z}}_{i}(x_{i},y_{i})\right]}^{2}\to min$$

(A5) $$\left\{ \begin{array}{l} na_{0}+a_{1}\sum_{i=1}^{n}x_{1i}+\cdots +a_{p}\sum_{i=1}^{n}x_{pi}=\sum_{i=1}^{n}z_{i}\\ a_{0}\sum_{i=1}^{n}x_{1i}+a_{1}\sum_{i=1}^{n}x_{1i}x_{1i}+\cdots +a_{p}\sum_{i=1}^{n}x_{pi}x_{1i}=\sum_{i=1}^{n}x_{1i}z_{i}\\ \vdots \\ a_{0}\sum_{i=1}^{n}x_{pi}+a_{1}\sum_{i=1}^{n}x_{1i}x_{pi}+\cdots +a_{p}\sum_{i=1}^{n}x_{pi}x_{pi}=\sum_{i=1}^{n}x_{pi}z_{i} \end{array}\right.$$

The matrix is

$$X=\left[\begin{array}{c} 1\\ 1\\ \vdots \\ 1 \end{array}\begin{array}{c} x_{11}\\ x_{12}\\ \vdots \\ x_{1n} \end{array}\begin{array}{c} x_{21}\\ x_{22}\\ \vdots \\ x_{2n} \end{array}\begin{array}{c} \cdots \\ \cdots \\ \cdots \end{array}\begin{array}{c} x_{p1}\\ x_{p2}\\ \vdots \\ x_{pn} \end{array}\right]\;Z=\left[\begin{array}{c} z_{1}\\ z_{2}\\ \vdots \\ z_{n} \end{array}\right]\;A=\left[\begin{array}{c} a_{0}\\ a_{1}\\ \vdots \\ a_{p} \end{array}\right]$$

Thus, Equation (A5) can be changed into

(A6) $$X^{T}XA=X^{T}Z$$

Taking the quadratic trend surface model $z=a_{0}+a_{1}x+a_{2}y+a_{3}x^{2}+a_{4}xy+a_{5}y^{2}$ as an example, its matrix is

$$\left[\begin{array}{c} \begin{array}{c} \begin{array}{c} 1\\ x_{1} \end{array}\\ y_{1} \end{array}\\ {x_{1}}^{2}\\ x_{1}y_{1}\\ {y_{1}}^{2} \end{array}\begin{array}{c} \begin{array}{c} \begin{array}{c} 1\\ x_{2} \end{array}\\ y_{2} \end{array}\\ {x_{2}}^{2}\\ x_{2}y_{2}\\ {y_{2}}^{2} \end{array}\begin{array}{c} \begin{array}{c} \begin{array}{c} \cdots \\ \cdots \end{array}\\ \cdots \end{array}\\ \cdots \\ \cdots \\ \cdots \end{array}\begin{array}{c} \begin{array}{c} \begin{array}{c} 1\\ x_{n} \end{array}\\ y_{n} \end{array}\\ {x_{n}}^{2}\\ x_{n}y_{n}\\ {y_{n}}^{2} \end{array}\right]*\left[\begin{array}{c} \begin{array}{c} \begin{array}{c} 1\\ 1 \end{array} \end{array}\\ \vdots \\ 1 \end{array}\begin{array}{c} \begin{array}{c} \begin{array}{c} x_{1}\\ x_{2} \end{array} \end{array}\\ \vdots \\ x_{n} \end{array}\begin{array}{c} \begin{array}{c} \begin{array}{c} y_{1}\\ y_{2} \end{array} \end{array}\\ \vdots \\ y_{n} \end{array}\begin{array}{c} \begin{array}{c} \begin{array}{c} {x_{1}}^{2}\\ {x_{2}}^{2} \end{array} \end{array}\\ \vdots \\ {x_{n}}^{2} \end{array}\begin{array}{c} \begin{array}{c} \begin{array}{c} x_{1}y_{1}\\ x_{2}y_{2} \end{array} \end{array}\\ \vdots \\ x_{n}y_{n} \end{array}\begin{array}{c} \begin{array}{c} \begin{array}{c} {y_{1}}^{2}\\ {y_{2}}^{2} \end{array} \end{array}\\ \vdots \\ {y_{n}}^{2} \end{array}\right]*\left[\begin{array}{c} \begin{array}{c} \begin{array}{c} a_{0}\\ a_{1} \end{array}\\ a_{2} \end{array}\\ a_{3}\\ a_{4}\\ a_{5} \end{array}\right]=\left[\begin{array}{c} \begin{array}{c} \begin{array}{c} 1\\ x_{1} \end{array}\\ y_{1} \end{array}\\ {x_{1}}^{2}\\ x_{1}y_{1}\\ {y_{1}}^{2} \end{array}\begin{array}{c} \begin{array}{c} \begin{array}{c} 1\\ x_{2} \end{array}\\ y_{2} \end{array}\\ {x_{2}}^{2}\\ x_{2}y_{2}\\ {y_{2}}^{2} \end{array}\begin{array}{c} \begin{array}{c} \begin{array}{c} \cdots \\ \cdots \end{array}\\ \cdots \end{array}\\ \cdots \\ \cdots \\ \cdots \end{array}\begin{array}{c} \begin{array}{c} \begin{array}{c} 1\\ x_{n} \end{array}\\ y_{n} \end{array}\\ {x_{n}}^{2}\\ x_{n}y_{n}\\ {y_{n}}^{2} \end{array}\right]*\left[\begin{array}{c} \begin{array}{c} \begin{array}{c} z_{0}\\ z_{1} \end{array} \end{array}\\ \vdots \\ z_{n} \end{array}\right],$$

i.e.,

(A7) $$A={\left(X^{T}X\right)}^{-1}X^{T}Z$$

The moving weighted trend surface analysis depends on the distance between two points, when each unknown point $\left(x_{i},y_{i}\right)$ interpolates variable Z. The closer the two points (estimated point and reference point) are to each other, the greater the weight.

(A8) $$p_{i}=\frac{1}{Si}$$

(A9) $$p_{i}=[\frac{\left(R-Si\right)}{Si}{]}^{2}$$

(A10) $$p_{i}=e^{-\frac{s_{i}^{2}}{R^{2}}}$$

where $p_{i}$ is the weight, $S_{i}$ is the distance between two points, and R is the sampling circle radius. For the terrain in this study, we chose Equation (A9). The matrix is:

$$P=\left[\begin{array}{c} \begin{array}{c} p_{1}\\ 0 \end{array}\\ \vdots \\ \vdots \\ 0 \end{array}\begin{array}{c} \begin{array}{c} 0\\ p_{2} \end{array}\\ \cdots \end{array}\begin{array}{c} \begin{array}{c} \cdots \end{array}\\ \ddots \\ \cdots \end{array}\begin{array}{c} \begin{array}{c} \cdots \end{array}\\ p_{n-1}\\ 0 \end{array}\begin{array}{c} 0\\ \begin{array}{c} 0\\ p_{n} \end{array} \end{array}\right].$$

Therefore,

(A11) $$A={\left(X^{T}PX\right)}^{-1}X^{T}PZ$$

In this study, we used the goodness of fit (R^2^) and F test with a 95% confidence level to evaluate different orders of polynomial regression equations to select the most suitable moving weighted trend surface model.

## Appendix B. Mann-Kendall Trend Test

The Mann-Kendall test is used to detect trends that are monotonic but not necessarily linear. The null hypothesis is independent and randomly ordered data in the Mann-Kendall test. The Mann-Kendall test statistic S is computed, as follows:

(A12) $$S=\sum_{i=1}^{n-1}\sum_{j=i+1}^{n}sgn(x_{j}-x_{i})$$

where $x_{j}$ and $x_{i}$ are the values in years j and i, j>i, respectively, and n is the number of data points. The value of $sgn(x_{j}-x_{i})$ is computed, as follows:

(A13) $$sgn(x_{j}-x_{i})=\left\{ \begin{array}{l} 1,\;if(x_{j}-x_{i})>0\\ 0,\;if(x_{j}-x_{i})=0\\ -1,\;if(x_{j}-x_{i})<0 \end{array}\right..$$

The test is conducted using a normal approximation, Z statistics, with the mean and the variance, as follows:

(A14) E(S)=0

(A15) $$Var(S)=\frac{1}{18}\left[n(n-1)(2n+5)-\sum_{p=1}^{q}t_{p}(t_{p}-1)(2t_{p}+5)\right]$$

where q is the number of tied (zero difference between compared values) groups, and $t_{p}$ is the number of data values in the $p^{th}$ group. The statistics S and Var(S) are used to compute Z, as follows:

(A16) $$Z=\left\{ \begin{array}{cc} \frac{S-1}{\sqrt{Var(S)}}, & S>0\\ 0, & S=0\\ \frac{S+1}{\sqrt{Var(S)}}, & S<0 \end{array}\right.$$

The Z value is used to evaluate the statistically significant trend, which is a positive value indicating an upward trend or a negative value indicating a downward trend, at the given significance levels.

## Appendix C. Standard Deviation Ellipse Analysis

Based on the average center of a set of discrete points, the standard deviation ellipse summarizes the spatial characteristics of geographic features (central tendency, dispersion, and directional trends). The method calculates the standard distances of other points from the average center and results in an ellipse with two directions that describe the overall characteristics of geospatial distributions. Therefore, the calculated multiyear standard deviation ellipses indicate clear trends in the directionality of the factors over time. The major and minor axes of the ellipses indicate the directions and ranges of the data distributions. Azimuths convey main trend directions, and changes in azimuth can reflect spatial rotations of overall factors in a certain direction. A median center, the center of all the data, identifies the location that minimizes the overall Euclidean distance to the features in a dataset and coincides with the arithmetic mean center under small variability. Herein, we used this method to trace the changes in spatial patterns of populations. Due to data availability limitations, the population data are only from 1990–2015. The algorithm is as follows:

The standard distances in the *x* and *y* directions are:

(A17) $$SDE_{x}=\sqrt{\frac{\sum_{i=1}^{n}{\left(x_{i}-x_{mc}\right)}^{2}}{n}}$$

(A18) $$SDE_{y}=\sqrt{\frac{\sum_{i=1}^{n}{\left(y_{i}-y_{mc}\right)}^{2}}{n}}$$

where $x_{i}$ and $y_{i}$ are the coordinates for feature i, $\left\{x_{mc},y_{mc}\right\}$ represents the mean center for the features, and n is the number of features.

The azimuth is calculated as follows:

(A19) $$tan\theta =\frac{A+B}{C}$$

(A20) $$A=(\sum_{i=1}^{n}x{x_{i}}^{2}-\sum_{i=1}^{n}y{y_{i}}^{2})$$

(A21) $$B=\sqrt{{\left(\sum_{i=1}^{n}x{x_{i}}^{2}-\sum_{i=1}^{n}y{y_{i}}^{2}\right)}^{2}+4{\left(\sum_{i=1}^{n}xx_{i}yy_{i}\right)}^{2}}$$

(A22) $$C=2\sum_{i=1}^{n}xx_{i}yy_{i}$$

where $xx_{i}$ and $yy_{i}$ are the deviations of the x and y coordinates from the mean center.

The standard deviations for the *x*-axis and *y*-axis are as follows:

(A23) $$\sigma_{x}=\sqrt{\frac{\sum_{i=1}^{n}{\left(xx_{i}\cos\theta -yy_{i}\sin\theta \right)}^{2}}{n}}$$

(A24) $$\sigma_{y}=\sqrt{\frac{\sum_{i=1}^{n}{\left(xx_{i}\sin\theta -yy_{i}\cos\theta \right)}^{2}}{n}}$$

## Appendix D. Dynamic ESV Assessment Method

By modifying and developing the method of Costanza et al. (1997) and Xie et al. (2015) of equivalence factor per unit area, Xie et al. (2017) initiated a dynamic assessment method for ESV. This method defines the spatiotemporal dynamic equivalence factors using net primary productivity (NPP), precipitation, and soil conservation per unit area as in Equations (25)–(28):

(A25) $$\left\{ \begin{array}{l} F_{nij}=P_{ij}*F_{n1}\;or\\ F_{nij}=R_{ij}*F_{n2}\;or\\ F_{nij}=S_{ij}*F_{n3} \end{array}\right.$$

(A26) $$P_{ij}=\frac{B_{ij}}{\bar{B}}$$

(A27) $$R_{ij}=\frac{W_{ij}}{\bar{W}}$$

(A28) $$S_{ij}=\frac{E_{ij}}{\bar{E}}$$

where $F_{nij}$ is the dynamic equivalence factor per unit area for ecosystem service n of an ecosystem in region i in month j; $P_{ij}$ is the spatiotemporal regulation factor of NPP in region i in month j; $R_{ij}$ is the spatiotemporal regulation factor of precipitation in region i in month j; $S_{ij}$ is the spatiotemporal regulation factor of soil conservation in region i in month j; $F_{n1}$ is the equivalence coefficient for the services from food production, raw materials supply, gas regulation, climate regulation, waste regulation, biodiversity maintenance and aesthetic landscape provision; $F_{n2}$ is the equivalence coefficient for the service from hydrology regulation; $F_{n3}$ is the equivalence coefficient for the service from soil conservation; $B_{ij}$ is the NPP of the ecosystem in region i in month j; $\bar{B}$ is the national annual NPP of the ecosystem; $W_{ij}$ is the average precipitation per unit area in region i in month j; $\bar{W}$ is the annual average precipitation per unit area; $E_{ij}$ is the quantity of soil conservation of the ecosystem in region i in month j; and, $\bar{E}$ is the average annual quantity of soil conservation per unit area.
