# Supplementary material for: Spatiotemporal Variation Characteristics of Ecosystem Service Losses in the Agro-Pastoral Ecotone of Northern China
Source: Int J Environ Res Public Health. 2019 Apr 3;16(7):1199. doi: 10.3390/ijerph16071199 (PMC6479984; doi:10.3390/ijerph16071199)
Supplement: Supplementary file 1 [file ijerph-16-01199-s001.pdf]

Supplementary Material

Spatiotemporal Variation Characteristics of Ecosystem Service Losses in the Agro-Pastoral Ecotone of Northern China

Yuejuan Yang, Kun Wang, Di Liu, Xinquan Zhao, Jiangwen Fan, Jinsheng Li, Xiajie Zhai, Cong Zhang and Ruyi Zhan

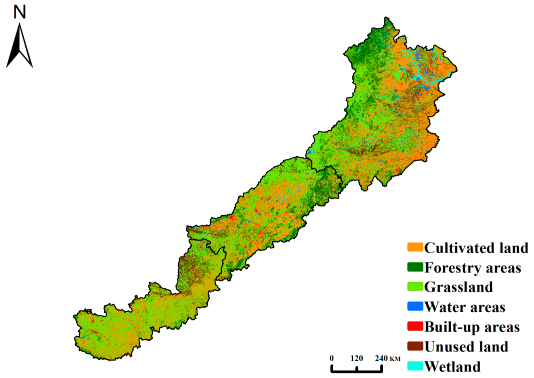

Figure S1. Land-use/land-cover (LULC) of the study area in 2015.

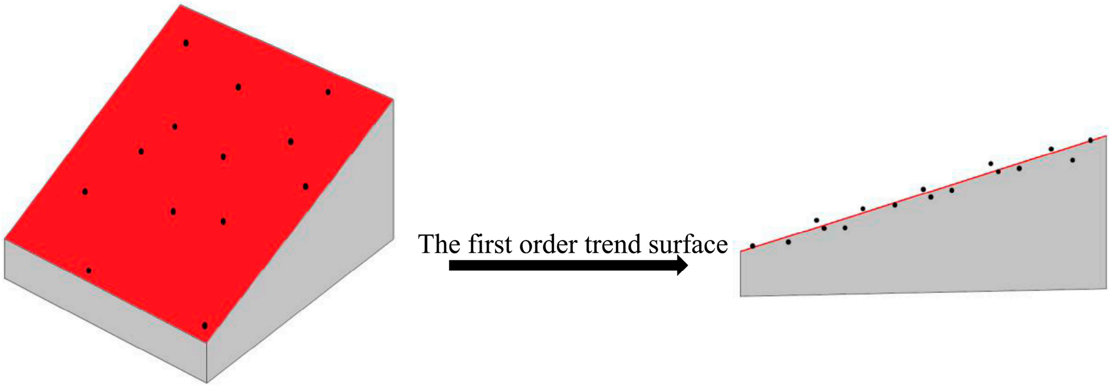

Figure S2. The schematic diagram of the first order trend surface.

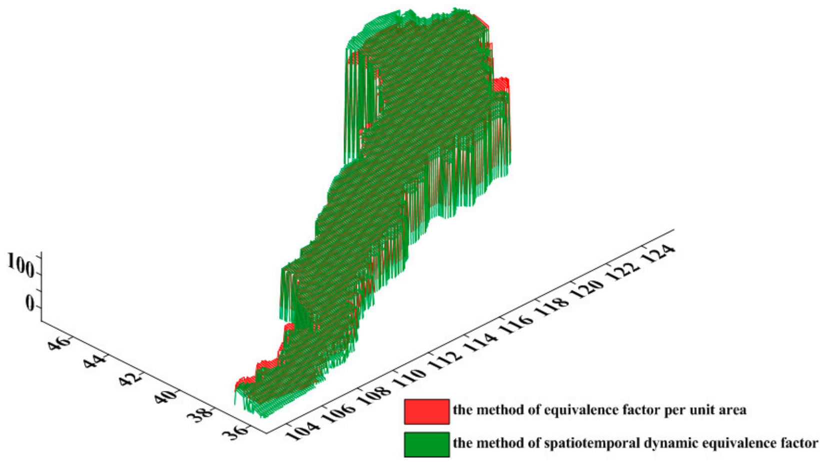

Figure S3. The result of evaluating the ESV losses in 2010–2015.

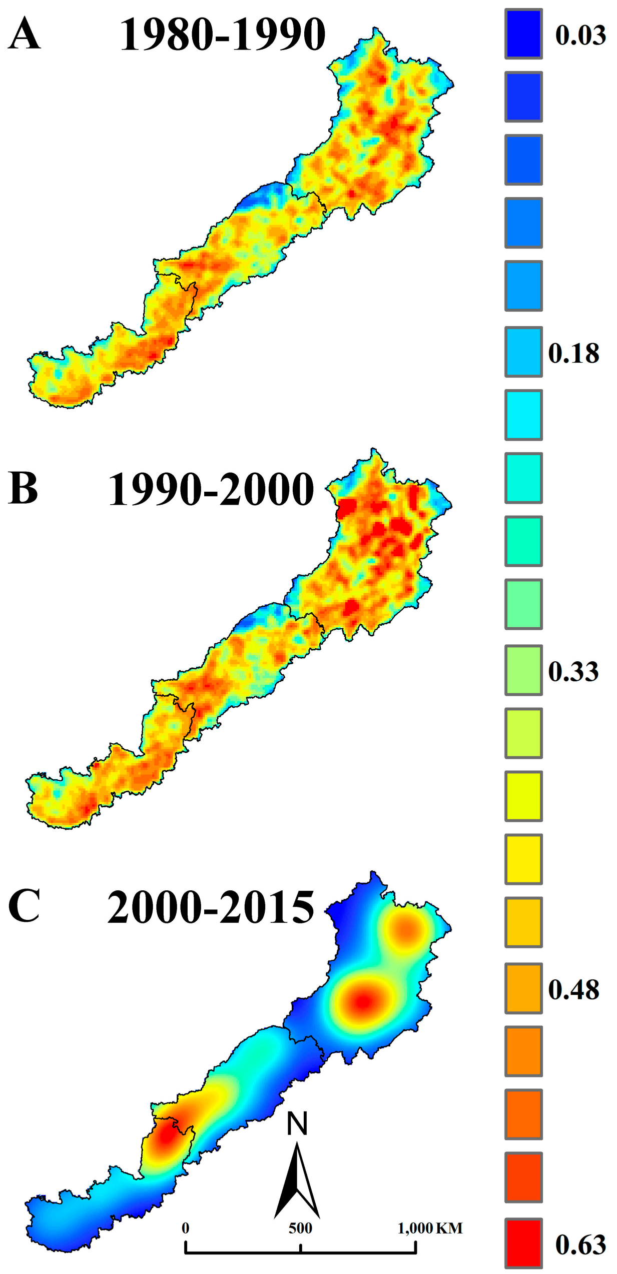

Figure S4. Spatial distribution of hotspots of ESV losses in 1980–1990 (**A**), 1990–2000 (**B**) and 2000–2015 (**C**).

Table S1. LULC classification.

Original primary land-use types	Original land-use subtypes	New primary land-use types	New land-use subtypes
Cultivated land	Paddy land	Cultivated land	Paddy land
	Dry land		Dry land
Forestry areas	Forest	Forestry areas	Forest
	Shrub		Shrub
	Woods		Woods
	Others		Others
Grassland	Dense grass	Grassland	Dense grass
	Moderate grass		Moderate grass
	Sparse grass		Sparse grass
Water areas	Stream and rivers	Water areas	Stream and rivers
	Lakes		Lakes
	Reservoir and ponds		Reservoir and ponds
	Permanent ice and snow	Built-up areas	Urban built-up areas
	Beach and shore		Rural settlements
	Bottomland		Others
Built-up areas	Urban built-up areas	Unused land	Sandy land
	Rural settlements		Gobi
	Others		Salina
Unused land	Sandy land		Bare soil
	Gobi		Bare rock
	Salina		Others
	Swampland	Wetland	Permanent ice and snow
	Bare soil		Beach and shore
	Bare rock		Bottomland
	Others		Swampland

Note: The original land use primary types and subtypes from Liu et al. (2005)

Table 2. Equivalence factor per unit area of ESV in China ($/ha × year based on the 2007 value of USD).

Type	Subtype	Cultivated land	Forestry areas	Grassland	Water areas	Unused land	Wetland
Provision	FP	57.5	18.98	24.73	30.48	1.15	20.7
	RMP	22.43	171.36	20.7	20.13	2.3	13.8
Regulation	GR	41.4	248.41	86.25	29.33	3.45	138.58
	CR	55.78	234.04	89.71	118.46	7.48	779.17
	HR	44.28	235.19	87.4	1079.34	4.03	772.84
	WT	79.93	98.91	75.9	853.92	14.95	828.05
Support	SC	84.53	231.16	128.81	23.58	9.78	114.43
	MB	58.65	259.34	107.53	197.24	23	212.19
Culture	ALP	9.78	119.61	50.03	255.31	13.8	269.69
Total		454.28	1616.99	671.06	2607.77	79.93	3149.45

Note: FP, food production; RMP, raw material production; GR, gas regulation; CR, climate regulation; HR, hydrology regulation; WT, waste regulation; SC, soil conservation; MB, biodiversity maintenance; ALP, aesthetic landscape provision.

Table S3. Parameters of the first order trend surface fitting.

	R^2	F	P	RSS	ESS
1980–1990	0.58	120.05	< 0.05	921032.91	1263659.06
1990–2000	0.60	131.20	< 0.05	1027830.82	1541163.21
2000–2015	0.51	89.13	< 0.05	17809240.04	18141303.12

Notes: RSS, residual sum of squares; ESS, explained sum of squares.
